# ASO Author Reflections: Closed-Loop Hypothermic Perfusion With ECMO Support: Expanding the Boundaries of Complex Liver Resection

**DOI:** 10.1245/s10434-025-18447-7

**Published:** 2025-10-04

**Authors:** Cornelius van Beekum, Moritz Schmelzle

**Affiliations:** General, Visceral and Transplant Surgery, Hannover Medical School, Hannover, Germany

Resection of centrally located liver tumors involving the hepatocaval confluence has long been associated with prohibitive risks. Total vascular exclusion (TVE) provides technical access but carries the burden of ischemia-reperfusion injury, systemic intestinal and renal venostasis, and hemodynamic instability. Previous strategies, such as ex situ or ante situm resection under uncontrolled hypothermic perfusion, have attempted to address these limitations, but are associated with relevant morbidity and mortality. A standardized, reproducible, and controlled strategy for protecting the future liver remnant in situ under TVE has been lacking.

In our recently accepted article ^1^, we report the Hannover closed-loop in situ hypothermic oxygenated perfusion (CLIP) concept combined with veno-venous extracorporeal membrane oxygenation (vvECMO). This dual approach establishes two independent circuits, with ECMO ensuring systemic hemodynamic stability and venous decompression while the closed-loop perfusion provides continuous hypothermic oxygenation of the liver remnant without systemic cooling. By recirculating the perfusate in a sterile loop, the technique prevents intraoperative hypothermia and uncontrolled contamination of the peritoneal cavity. This innovation enables safe and oncologically radical resection of a centrally located intrahepatic cholangiocarcinoma previously deemed unresectable.

Our work complements recent reports from other groups ^2^ exploring transplant-inspired strategies to mitigate ischemia-reperfusion injury in complex resections. Although technical variations exist, the shared objective is targeted protection of the liver remnant during TVE. ^3^ To move beyond single-center experiences, harmonization of methods and prospective clinical evaluation will be required. Multicenter collaborations should define optimal protocols, assess reproducibility, and identify patient populations most likely to benefit, ultimately expanding the boundaries of safe resection in hepatobiliary oncology.
